# CGRP-monoclonal antibodies in Japan: insights from an online survey of physician members of the Japanese headache society

**DOI:** 10.1186/s10194-024-01737-y

**Published:** 2024-03-15

**Authors:** Tsubasa Takizawa, Keiko Ihara, Narumi Watanabe, Ryo Takemura, Nobuyuki Takahashi, Naoki Miyazaki, Mamoru Shibata, Keisuke Suzuki, Noboru Imai, Norihiro Suzuki, Koichi Hirata, Takao Takeshima, Jin Nakahara

**Affiliations:** 1 Department of Neurology, Keio University School of Medicine, 35 Shinanomachi, Shinjuku-ku, Tokyo, 160-8582 Japan; 2 Japanese Red Cross Ashikaga Hospital, Ashikaga, Japan; 3 Biostatistics Unit, Clinical and Translational Research Center, Keio University Hospital, Tokyo, Japan; 4 Department of Neurology, Tokyo Dental College Ichikawa General Hospital, Chiba, Japan; 5 Department of Neurology, Dokkyo Medical University, Mibu, Japan; 6 Department of Neurology and Headache Center, Japanese Red Cross Shizuoka Hospital, Shizuoka, Japan; 7 Department of Neurology, Shonan Keiiku Hospital, Fujisawa, Japan; 8 Headache Center, Department of Neurology, Tominaga Hospital, Osaka, Japan; 9 Task Force for the Use of Anti-CGRP Monoclonal Antibodies, The Japanese Headache Society, Tokyo, Japan

**Keywords:** CGRP monoclonal antibody, Migraine, Japanese Headache Society, Guideline, Online survey, Cost

## Abstract

**Background:**

Anti-calcitonin gene-related peptide monoclonal antibodies (CGRPmAbs) have greatly changed migraine treatment options. In Japan, although CGRPmAb guidelines (≥ 4 monthly migraine days (MMDs) and ≥ 1 previous preventive failure) are well-acknowledged, the actual use of CGRPmAbs and the circumstances of the related headache care are unknown.

**Methods:**

We conducted an online survey of Japanese Headache Society members, inquiring about the physicians' experience with CGRPmAbs and how they make decisions related to their use.

**Results:**

Of the 397 respondents, 320 had prescribed CGRPmAbs. The threshold number of previous preventive failures for recommending a CGRPmAb was two for the majority of the respondents (*n* = 170, 54.5%), followed by one (*n* = 64, 20.5%). The MMD threshold was ≥ 4 for 71 respondents (22.8%), ≥ 6 for 68 (21.8%), ≥ 8 for 76 (24.4%), and ≥ 10 for 81 (26.0%). The respondents tended to assess treatment efficacy after 3 months (episodic migraine: *n* = 217, 69.6%, chronic migraine: *n* = 188, 60.3%). The cost of CGRPmAbs was described by many respondents in two questions: (*i*) any request for a CGRPmAb (27.7%), and (*ii*) the most frequently reported reason for responders to discontinue CGRPmAbs (24.4%).

**Conclusions:**

Most of the respondents recommended CGRPmAbs to patients with ≥ 2 preventive failures, followed by ≥ 1. The MMD threshold ranged mostly from ≥ 4 to ≥ 10. The concern for costs was raised as a major limiting factor for prescribing CGRPmAbs.

**Supplementary Information:**

The online version contains supplementary material available at 10.1186/s10194-024-01737-y.

## Background

Migraine is a common headache disorder with a worldwide prevalence of approximately 14% [1] and a specific prevalence of approximately 8.4% in Japan [2]. Conventional preventive treatments for migraines include anticonvulsants, antidepressants, beta-blockers, and calcium channel blockers. However, these treatments have proven problematic, with limited efficacy and numerous adverse effects. The advent of anti-calcitonin gene-related peptide monoclonal antibodies (CGRPmAbs) has revolutionized migraine preventive treatments [3–5].

CGRPmAbs received approval in Japan in 2021, and galcanezumab was launched in April 2021, followed by fremanezumab and erenumab in August 2021. Several CGRPmAbs have shown efficacy and safety in clinical trials [6–9] and have also been demonstrated to be effective in real-world studies [10, 11], improving the QOL of patients with migraine. Importantly, CGRPmAbs have demonstrated a faster onset of efficacy and are associated with only a few minor adverse effects.

The Japanese Headache Society (JHS) recommendations for prescribing a CGRPmAb to a patient with migraine include two thresholds: ≥ 4 monthly migraine days (MMDs) and ≥ 1 preventive failure. In addition, CGRPmAbs can only be prescribed at facilities that have ≥ 1 physician in charge who have ≥ 5 years of experience treating headaches and have at least one board certification in the fields of headache, neurology, neurosurgery, or internal medicine. Physicians with < 5 years of experience who work at such facilities can also prescribe CGRPmAbs under the guidance of the responsible physician. If a patient is indicated for a CGRPmAb, each dose of CGRPmAb will require a co-pay of approximately 13,000 JPY (equivalent to 83 EUR or 91 USD as of December 23, 2023), which is 30% of the CGRPmAbs' original cost; the rest of the cost is covered by Japan's national health insurance program.

Though the JHS guideline has been widely appreciated by headache specialists, given the recent evidence suggesting CGRPmAbs as early intervention [12], detailed information on CGRPmAbs use and actual circumstances of headache care is needed to further optimize it. To obtain this information, we distributed a survey to the members of the JHS about their use of CGRPmAbs in their daily practices.

## Methods

### Ethics

This study was approved by the Ethics Committee of the Keio University School of Medicine (approval no. 20221100). Participants were informed about the purpose and the content of the study through the online survey form. The need for informed consent was waived by the Ethics Committee of the Keio University School of Medicine in accordance with national regulations (Ethical Guidelines for Medical and Biological Research Involving Human Subjects).

### Collected items

We delivered an online survey via email on December 22, 2022 to the physician membership of the JHS, which included approximately 3,000 physicians and 1,000 board-acquired headache specialists at that time. The physicians were asked to complete the survey by January 22, 2023. The timing of the survey was 1 year and 8 months after the launch of the first CGRPmAb in Japan, i.e., galcanezumab. As presented in Supplementary Table S1, the survey collected information including the following: the respondent's type of facility, service, age, years of headache practice, years of JHS membership, board certification(s), the average length of the first appointment for the headache patients (in minutes), the average length of the follow-up appointments for the headache patients (in minutes), the number of patients with migraine who were regularly followed up, the number of patients with migraine who had ever used migraine preventive treatments, the number of patients with migraine who had ever been treated with a CGRPmAb, the experience using CGRPmAbs, the availability of CGRPmAbs at each facility, the threshold of monthly migraine days (MMDs) for recommending a CGRPmAb, the number of migraine preventives usually tried before a CGRPmAb was prescribed, the point at which to assess the response to a CGRPmAb for patients with episodic migraines (EM) and for patients with chronic migraines (CM), the percentages of EM and CM patients whose MMDs had decreased by ≥ 50%, the most frequently reported reason for CGRPmAb-responders to discontinue CGRPmAbs, and any requests related to CGRPmAbs.

The MMD threshold for recommending a CGRPmAb and the number of migraine preventives usually tried before a CGRPmAb was prescribed were of particular interest, and we analyzed other outcomes as well.

### Data analysis

We summarized each variable using mean ± standard deviation (median [first quartile, third quartile]) for continuous variables and the frequency (percentage) for categorical variables. We used Student's t-test for two-group comparison and an analysis of variance (ANOVA) for multiple-group comparison in parametric continuous variables and Kruskal-Wallis test in non-parametric continuous variables. Fisher's exact test was used for the assessment of between-group differences in categorical variables. Statistical significance was set at *p* < 0.05. The statistical analyses were conducted with SAS ver. 9.4 software (SAS Institute, Cary, NC, USA). We allowed the respondents to submit only completed responses, and we thus did not impute any missing data. We excluded survey answers that were considered inexplicable for the analysis (i.e., answering > 600 min for the duration of the interview, or the number of patients treated with a CGRPmAb was the same or greater than the number of patients with migraine).

## Results

A total of 400 physician members of the JHS responded to the survey, and the responses of 397 of the physicians were included in the analyses; the other three physicians' answers were deemed inappropriate for the analyses. Another nine responses were partly excluded from the analysis since they included inexplicable responses regarding the number of patients.

**Fig. 1:**
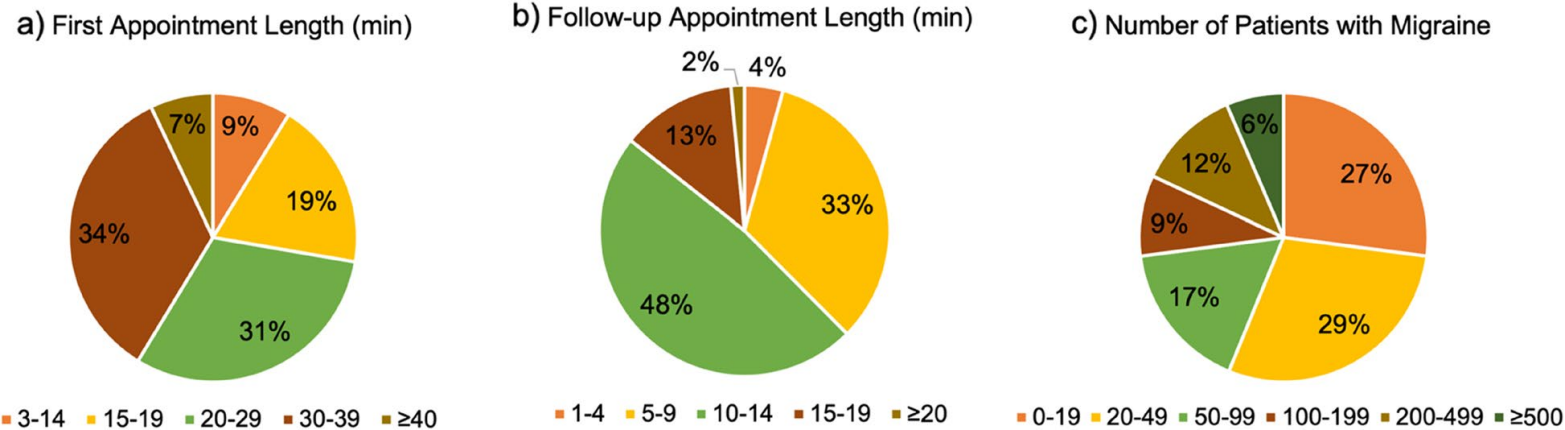
Appointment length and number of patients who are followed up regularly. **a** Length of the headache patients' first appointments (min). **b** Length of the headache patients' follow-up appointments (min). **c** No. of patients with migraine who are followed up regularly

The average length of the first appointments for the headache patients was 23.5 ± 9.5 (20 [15, 30]) min, and the average length of the follow-up appointments was 9.0 ± 3.9 (10 [5, 10]) min (Table 1). As depicted in Fig. 1, while 91% of them spent ≥ 15 min for the first appointment (15–19 min: 19%, 20–29 min: 31%, 30–39 min: 34%, ≥ 40: 7%), 85% of them spent < 15 min for the follow-up appointments (1–4 min: 4%, 5–9 min: 33%, 10–14 min: 48%). The physicians regularly followed up 121.8 ± 263.7 (30 [15, 100]) patients with migraines (0–19: 27%, 20–49: 29%, 50–99: 17%, 100–199: 9%, 200–499: 12%, ≥ 500: 6%), of whom 77.1 ± 165.7 (20 [9, 67.5]) patients had ever used a migraine preventive treatment and 25.5 ± 45.9 (10 [4, 25]) had ever been treated with a CGRPmAb (Table 1). The responses also revealed that 80.6% (*n* = 320) of the physicians had experience prescribing CGRPmAbs. The ratio of patients who had received a migraine preventive treatment over the total number of migraine patients was 64.9 ± 23.2%. The ratio of patients who had received a CGRPmAb as a migraine preventive treatment over the total number of migraine patients was 23.9 ± 17.1%. These data also revealed that the ratio of patients treated with a CGRPmAb over the total number of patients who received a migraine preventive treatment was 37.6 ± 26.1%.

**Table 1:** General information about headache practice

**Item**	mean ± standard deviation	median [Q1, Q3]
Length of the headache patients' first appointments, min	23.5 ± 9.5	20 [15, 30]
Length of the headache patients' follow-up appointments, min	9.0 ± 3.9	10 [5, 10]
No. of patients with migraine who are followed up regularly^a^	121.8 ± 263.7	30 [15, 100]
No. of patients with migraine who are followed up regularly and have ever used preventive treatments^a^	77.1 ± 165.7	20 [9, 67.5]
Any experience using CGRPmAbs	Yes: 320 (80.6%)	
No. of patients with migraine who are followed up regularly and have ever used CGRPmAbs^b^	25.5 ± 45.9	10 [4, 25]

The data are presented as mean ± standard deviation and median [first quartile (Q1), third quartile (Q3)] (*n* = 397)

^a^Nine answers were excluded (*n* = 388)

^b^Eight answers were excluded (*n* = 312). CGRPmAb: anti-calcitonin gene-related peptide monoclonal antibody, min: minute, No.: number

The types of facilities where the physician respondents were working were mainly community hospitals (*n* = 194, 48.9%), followed by clinics (*n* = 126, 31.7%) and university hospitals (*n* = 77, 19.4%) (Table 2). Most of the respondents worked in either neurology (*n* = 170, 42.8%) or neurosurgery (*n* = 185, 46.6%) services, but there were also physicians in the fields of anesthesiology/pain clinic (*n* = 16, 4.0%), internal medicine (*n* = 9, 2.3%), pediatrics (*n* = 9, 2.3%), and others. Regarding age, most of the respondents were > 40 years old (*n* = 362, 91.2%). They were also well experienced in migraine treatment, with only 24 (6.0%) of the respondents having < 5 years of experience treating headaches. Moreover, about half of the respondents (*n* = 202, 50.9%) had ≥ 10 years of JHS membership. Only 23 respondents (5.8%) did not have any board certifications at the time of the survey (Table 2).

**Table 2:** Demographic data of the respondents

Item	Answer	frequency, %(*n* = 397)
Facility	Clinic	126, 31.7%
	Community hospital	194, 48.9%
	University hospital	77, 19.4%
Service	Neurology	170, 42.8%
	Neurosurgery	185, 46.6%
	Internal medicine (other than neurology)	9, 2.3%
	Pediatrics	9, 2.3%
	Anesthesiology/Pain clinic	16, 4.0%
	Rehabilitation	1, 0.3%
	Ob-Gyn	1, 0.3%
	Otorhinolaryngology	3, 0.8%
	Psychiatry	2, 0.5%
	General medicine	1, 0.3%
Age, yrs	20 s	3, 0.8%
	30 s	32, 8.1%
	40 s	118, 29.7%
	50 s	131, 33.0%
	60 s or older	113, 28.5%
Years of headache practice	< 5 yrs	24, 6.0%
	≥ 5 and < 10 yrs	38, 9.6%
	≥ 10 and < 20 yrs	125, 31.5%
	≥ 20 yrs	210, 52.9%
Years of JHS membership	< 1 yr	52, 13.1%
	≥ 1 and < 5 yrs	70, 17.6%
	≥ 5 and < 10 yrs	73, 18.4%
	≥ 10 yrs	202, 50.9%
Boards	Headache	232, 58.4%
	Neurology	164, 41.3%
	Neurosurgery	181, 45.6%
	Internal Medicine	99, 24.9%
	None	23, 5.8%
CGRPmAb that is available at your facility	Erenumab	244, 61.5%
	Galcanezumab	337, 84.9%
	Fremanezumab	272, 68.5%

*JHS* Japanese Headache Society, *CGRPmAb* Anti-calcitonin gene-related peptide monoclonal antibody, *Ob-Gyn* Obstetrics and gynecology, *yrs* years

In terms of CGRPmAb usage, the most widely available CGRPmAb was galcanezumab (*n* = 337, 84.9%), followed by fremanezumab (*n* = 272, 68.5%) and erenumab (*n* = 244, 61.5%) (Table 2). The number and types of CGRPmAbs available at each facility differed among the community hospitals, clinics, and university hospitals (Suppl. Table S2). All three CGRPmAbs, i.e., galcanezumab, fremanezumab, and erenumab, were available in more than half of the facilities. The percentage of facilities where all three CGRPmAbs were available was highest in the clinics (*n* = 85, 67.5%) (Suppl. Table S2).

The threshold for the number of MMDs for recommending a CGRPmAb was 10 for 81 (26.0%) of the respondents, and the number of migraine preventives usually tried before prescribing a CGRPmAb was two for 170 respondents (54.5%) followed by one for 64 respondents (20.5%) (Table 3). When the physician respondents were asked about the appropriate time point at which to assess the response to a CGRPmAb in patients with EM or CM, the response of 217 (69.6%) was after 3 months for EM patients; 188 (60.3%) specified after 3 months for CM patients. For patients with CMs, 55 (17.6%) of the physicians waited for 4–6 months before the assessment, whereas 19 (6.1%) of the physicians waited for 4–6 months in cases of EM (Table 3).

**Table 3:** Assessment of CGRPmAbs' suitability and efficacy

Item	Answer	frequency, % (*n* = 312)
The MMD threshold for recommending CGRPmAbs	≥ 4	71, 22.8%
	≥ 6	68, 21.8%
	≥ 8	76, 24.4%
	≥ 10	81, 26.0%
	≥ 12	4, 1.3%
	≥ 15	12, 3.8%
The number of migraine preventives you usually try before prescribing a CGRPmAb	1	64, 20.5%
	2	170, 54.5%
	3	62, 19.9%
	4	11, 3.5%
	≥ 5	5, 1.6%
When to assess the response to a CGRPmAb in patients with EM	After 1 month	46, 14.7%
	After 2 months	30, 9.6%
	After 3 months	217, 69.6%
	After 4–6 months	19, 6.1%
	After 7–9 months	0, 0%
	After 10–12 months	0, 0%
When to assess the response to a CGRPmAb in patients with CM	After 1 month	34, 10.9%
	After 2 months	30, 9.6%
	After 3 months	188, 60.3%
	After 4–6 months	55, 17.6%
	After 7–9 months	5, 1.6%
	After 10–12 months	0, 0%
The percentage of EM patients whose MMDs have decreased ≥ 50%	< 20%	7, 2.2%
	≥ 20% and < 40%	11, 3.5%
	≥ 40% and < 60%	85, 27.2%
	≥ 60% and < 80%	102, 32.7%
	≥ 80%	107, 34.3%
The percentage of CM patients whose MMDs have decreased ≥ 50%	< 20%	16, 5.1%
	≥ 20% and < 40%	53, 17.0%
	≥ 40% and < 60%	104, 33.3%
	≥ 60% and < 80%	66, 21.2%
	≥ 80%	73, 23.4%
The most frequently reported reason for responders to discontinue CGRPmAbs	Cost	76, 24.4%
	Adverse effects, safety (including injection site reaction, constipation, pregnancy)	39, 12.5%
	Frequency of hospital visits	6, 1.9%
	Enough improvement of migraine	169, 54.2%

*CM* Chronic migraine, *EM* Episodic migraine, *M* Months, *MMD* Monthly migraine day, *CGRPmAb* Anti-calcitonin gene-related peptide monoclonal antibody

Regarding efficacy, we asked the percentage of patients whose MMDs had decreased by ≥ 50% and defined it as the responder rate (RR). For EM patients, the RR was ≥ 80% as answered by 107 (34.3%) respondents. The RR in CM patients was ' ≥ 40% and < 60%,' with 104 (33.3%) responses. The most frequently reported reasons for CGRPmAb responders to discontinue treatment with a CGRPmAb were: (*i*) sufficient improvement of migraines (*n* = 169, 54.2%), (*ii*) high cost (*n* = 76, 24.4%), and (*iii*) adverse effects (*n* = 39, 12.5%) (Table 3). Our survey also asked whether the physicians had any requests about CGRPmAbs, and "cost of CGRPmAbs" was the most frequently mentioned request in their answers (*n* = 110, 27.7%), followed by "establishment of detailed treatment plans" (*n* = 13, 3.3%).

A comparison of the survey item responses between the physicians with experience prescribing CGRPmAbs and those without such experience revealed that in the group with experience, the length of follow-up appointments was slightly shorter (*p* = 0.012) and the number of patients who were followed up was significantly higher (*p* < 0.0001) (Table 4). The age, years of headache practice, and years of JHS membership also showed significantly different distributions between the experienced and not-experienced groups. The physicians with CGRPmAb experience also had a significantly higher rate of having at least one board certification (*p* < 0.0001) (Table 4).

**Table 4:** The difference in headache practice based on experiences using CGRPmAbs

Item		Experienced (*n* = 320)	Not experienced (*n* = 77)	*p*-value
Length of the patients' first appointments, min		23.6 ± 9.7	23.1 ± 8.5	0.6758
Length of the patients' follow-up appointments, min		8.8 ± 3.6	10.0 ± 4.7	0.012
No. of patients with migraine who are followed up regularly^a^		50 [20, 150]	10 [5, 20]	<0.0001
Age, yrs	20 s	2, 0.6%	1, 1.3%	0.0006
	30 s	19, 5.9%	13, 16.9%	
	40 s	87, 27.2%	31, 40.3%	
	50 s	113, 35.3%	18, 23.4%	
	≥ 60 s	99, 30.9%	14, 18.2%	
Years of headache practice	< 5 yrs	14, 4.4%	10, 13.0%	0.0004
	≥ 5 and < 10 yrs	24, 7.5%	14, 18.2%	
	≥ 10 and < 20 yrs	101, 31.6%	24, 31.2%	
	≥ 20 yrs	181, 56.6%	29, 37.7%	
Years of JHS membership	< 1 yrs	30, 9.4%	22, 28.6%	< 0.0001
	≥ 1 and < 5 yrs	49, 15.3%	21, 27.3%	
	≥ 5 and < 10 yrs	58, 18.1%	15, 19.5%	
	≥ 10 yrs	183, 57.2%	19, 24.7%	
Boards	≥ 1	311, 97.2%	63, 81.8%	<0.0001

^a^Eight and one answers were excluded from the 'Experienced' and 'Not experienced' groups, respectively. The data are presented as mean ± standard deviation or median [first quartile, third quartile]

*No* Number, *JHS* Japan Headache Society, *min* minute, *yrs* years, *CGRPmAb* Anti-calcitonin gene-related peptide monoclonal antibody

**Fig. 2:**
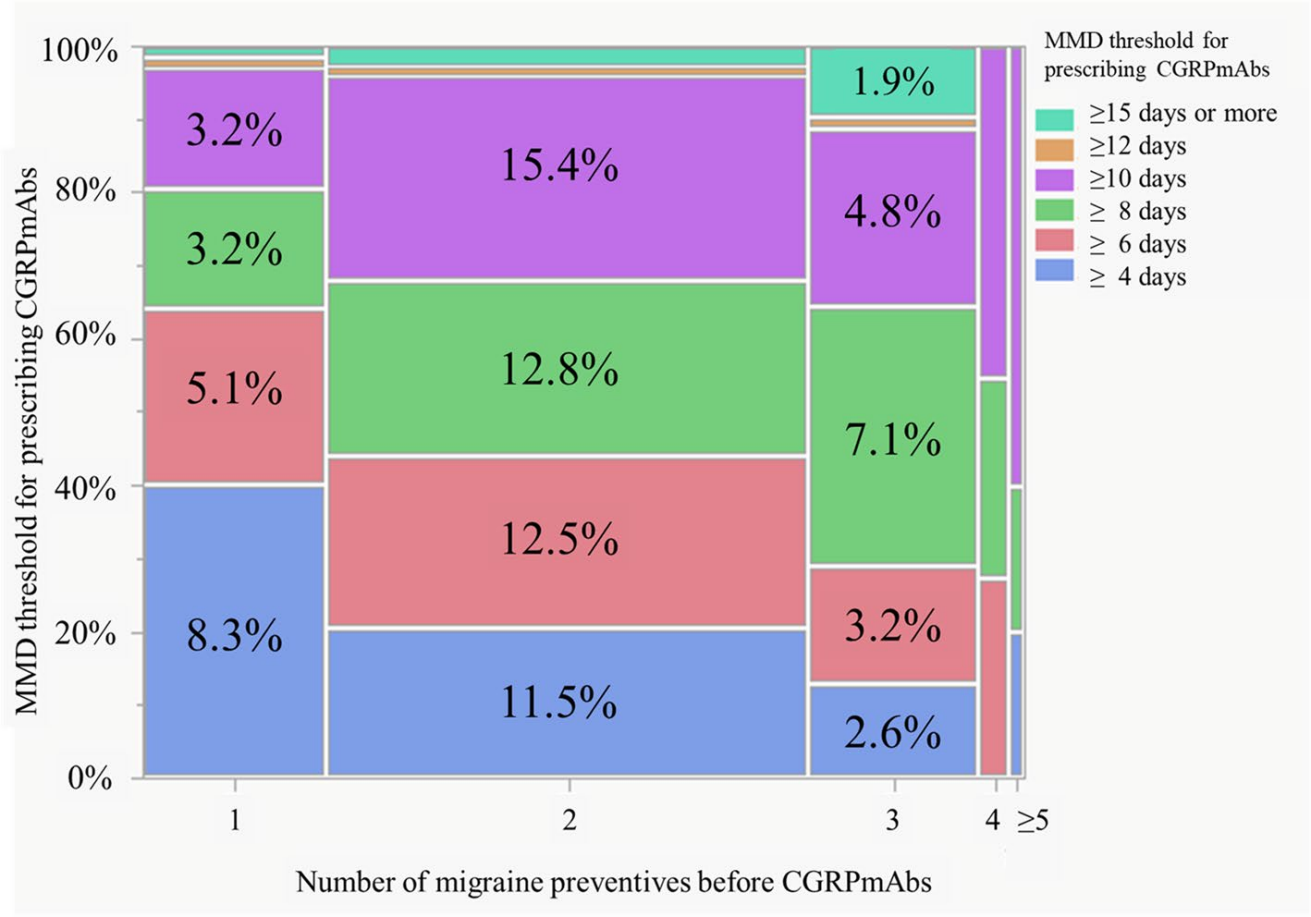
Number of migraine preventives before an anti-calcitonin gene-related peptide monoclonal antibody (CGRPmAb) is prescribed, and the monthly migraine days (MMD) threshold for prescribing a CGRPmAb

Focusing on how the physicians decided whether or not to prescribe a CGRPmAb to patients, we determined the MMD threshold for prescribing CGRPmAbs and the number of migraine preventives tried before CGRPmAbs (Suppl. Table S3). Although approximately half of the respondents answered 'two' for the number of migraine preventives attempted before CGRPmAbs (*n* = 170, 54.5%) followed by 'one' (*n* = 64, 20.5%), the MMD threshold for prescribing CGRPmAbs was rather dispersed among ' ≥ 4' (*n* = 71, 22.8%), ' ≥ 6' (*n* = 68, 21.8%), ' ≥ 8' (*n* = 76, 24.4%), and ' ≥ 10' (*n* = 81, 26.0%). Regarding the combination of these parameters, the MMDs of ' ≥ 10' and 'two' migraine preventives before the use of a CGRPmAb accounted for the highest proportion (*n* = 48, 15.4%), followed by ≥ 8 MMDs and two preventives (*n* = 40, 12.8%), ≥ 6 MMDs and two preventives (*n* = 39, 12.5%), and ≥ 4 MMDs and two preventives (*n* = 36, 11.5%) (Fig. 2).

We also analyzed the between-subgroup differences in the MMD threshold for prescribing a CGRPmAb, and we observed that the number of migraine patients who were followed up regularly (*p* = 0.0026) and the number of migraine patients who had ever been treated with a CGRPmAb (*p* = 0.0109) were related to the MMD threshold. Both the number of patients with migraine who were followed up regularly and the number of patients with migraine who were followed up regularly and had ever been treated with a CGRPmAb were the highest among the physician respondents who used the threshold of ≥ 8 MMDs (Table 5).

**Table 5:** The number of patients followed up categorized by the MMD threshold for prescribing a CGRPmAb

MMD threshold for prescribing CGRPmAbs	No. of patients with migraine who are followed up regularly			No. of patients with migraine who are followed up regularly and have ever used CGRPmAbs		
	***N***^**a**^	**Median [Q1, Q3]**	***p*-value**	***N***^**a**^	**Median [Q1, Q3]**	***p*-value**
≥ 4	71	30 [20, 100]	0.0026	71	10 [3, 30]	0.0109
≥ 6	68	35 [20, 100]		68	6 [3, 22.5]	
≥ 8	76	90 [30, 240]		76	14.5 [6, 50]	
≥ 10	81	50 [20, 100]		81	7 [3, 20]	
≥ 12	4	17.5 [12.5, 70]		4	4.5 [2.5, 22.5]	
≥ 15	12	56 [20, 155]		12	5.5 [3.5, 15.0]	

The data are presented as median [first quartile, third quartile]

*MMD* Monthly migraine days, *CGRPmAb* Anti-calcitonin gene-related peptide monoclonal antibody, *No* Number

^a^N indicates the number of physicians, Q1: first quartile, Q3: third quartile

## Discussion

The results of our online survey conducted 1 year and 8 months after the first CGRPmAb's launch in Japan demonstrated that (*i*) most of the physician respondents prescribed a CGRPmAb when patients with migraine had been treated with ≤ 2 preventives (1: 20.5%, 2: 54.5%), and (*ii*) the CGRPmAbs' efficacy was assessed after 3 months. The MMD threshold varied widely among the respondents, from ≥ 4 to ≥10 MMDs. The surveyed physicians with experience using CGRPmAbs were very well-trained in terms of the number of years of headache practice and JHS membership compared to the physicians who had no experience with CGRPmAb. Of note, there were only 14 physicians (4.4%) with < 5 years of headache practice and nine physicians (2.8%) without board certification; we suspect that these physicians were working with headache specialists who meet the criteria for prescribing CGRPmAbs and are responsible for patients' medical treatment.

The reimbursement policy and guidelines of CGRP mAbs have attracted attention since the recent report from Germany showed that a policy change to expand the use of erenumab has resulted in favorable outcomes for patients with migraine and suggested that real-world evidence on the use of CGRP mAbs has to be investigated further to optimize the use of CGRP mAbs [13]. Currently in Japan, CGRPmAbs are recommended for and reimbursed for patients who have experienced ≥ 4 MMDs and ≥ 1 preventive failure. We suspect that these criteria for reimbursement are very generous compared to the other countries. For example, in the U.S., the American Headache Society outlined the following indications for initiating treatment with a CGRPmAb: patients meeting the ICHD-3 (International Classification of Headache Disorders, third edition) criteria for migraine with or without aura who (*i*) have 4–7 monthly headache days with at least moderate disability, defined as (MIDAS [Migraine Disability Assessment] score > 11, Headache Impact Test [HIT]-6 score > 50), or (*ii*) have ≥ 8 monthly headache days, and intolerability or an inadequate response to a 6-week trial after taking at least two of the following preventive treatments: topiramate, divalproex sodium/valproate sodium, beta-blocker, tricyclic antidepressant, serotonin-norepinephrine reuptake inhibitor, two quarterly injections of onabolulinumtoxinA (for those with CM), and other Level A or B treatments (established efficacy or probably effective) according to the American Academy of Neurology/American Headache Society guidelines [14]. However, the coverage of a medication by insurance and the corresponding co-pay amount depends on the individual patient's medical insurance plans and the different reimbursement criteria set by individual insurance companies. The co-pay may range from $0 up to the full price. According to an American healthcare company GoodRx [15], the full price for self-injectable CGRPmAbs ranges from 600 USD to over 1000 USD. In Europe, though CGRPmAbs are now recommended as the first-line treatment option [12], the reimbursement criteria vary among countries. For example, in Spain, reimbursement is limited to patients with ≥ 8 MMD and ≥ 3 previous failures including onabotulinumtoxinA in CM [16]. In Denmark, CGRPmAbs are reimbursed for patients who have CM without medication-overuse headache (MOH) and have failed to achieve a sufficient response to preventive medication including ≥ 1 antihypertensive and ≥ 1 antiepileptic drug. The criteria are also strict in other countries in Asia, such as Taiwan, where the reimbursement is restricted to patients with CM with ≥ 3 failures of preventives including topiramate [17]. As long as the criteria are met, patients do not have to pay out of pocket due to the reimbursement in Spain, Denmark, or Taiwan.

Despite the very generous JHS guidelines for recommendation and reimbursement of CGRPmAbs in Japan, our present survey results revealed that most of the respondents had been using a reasonably limited threshold for recommending CGRPmAbs. This might be attributed to the financial burden, which was also the most frequently mentioned issue concerning CGRPmAbs, and/or to one or more other factors that were not addressed in our survey, such as sufficient management only with acute treatment or conventional migraine preventatives.

Our assessment of the respondents' answers regarding their patients' responses to CGRPmAbs showed that the majority of the respondents assessed the patient responses after 3 months of treatment, which is in accordance with real-world evidence obtained in Japan [10, 18]. Specifically for CM patients, 17.6% of the physicians assessed the response after 4–6 months. The 50% RRs based on this survey (EM: '≥ 80%', 107 [34.3%], ' ≥ 60% and < 80%,' 102 [32.7%]); CM: ' ≥ 40% and < 60%,' 104 [33.3%]) were also in the same range as the real-world evidence but in a higher range compared to clinical trials [6–9]. However, a recent study from Italy showed that some of the non-responders were actually late responders to CGRPmAbs [19], and this finding merits further investigation.

In terms of the cost of CGRPmAbs, we obtained two significant findings: (*i*) 27.7% of the respondents reported being concerned about the cost, and (*ii*) 24.4% of them identified cost as the most frequently reported reason for discontinuing treatment with a CGRPmAb. These findings suggest a need to optimize the pricing of CGRPmAbs in order to enhance access to them. As mentioned above, the use of CGRPmAbs in Japan usually incurs an expense of approximately 13,000 JPY (equivalent to 83 EUR or 91 USD as of December 23, 2023) per dose after the reimbursement, which is excessively high for some patients, given the average annual salary in Japan of 4,580,000 JPY (equivalent to 29,166 EUR or 32,164 USD as of December 23, 2023) [20].

To our understanding, this is the first report from Japan that sheds light on two unique aspects of headache practice in Japan. First, we suspect that the duration of each patient appointment in Japan is much shorter compared to most of the other countries, with first and follow-up appointments being < 25 min and < 10 min on average, respectively. The physicians with CGRPmAb experience reported regularly following up about 150 patients. Second, not only neurologists but also many neurosurgeons in Japan have been treating migraines with CGRPmAbs.

As part of the JHS, we surveyed the real-world use of CGRPmAbs in Japan and suggest further investigation in other regions. We propose that similar surveys be conducted internationally with similar methods as the previous survey [21], which would potentially yield insightful results on the uses of CGRPmAbs. To the best of our knowledge, the present study is the first to clarify the duration of each appointment and the number of patients followed up by each physician. Given that other migraine therapies will be available in the upcoming years and guidelines for headache diagnosis and treatment will be updated constantly, it is important to capture the current real-world situation of headache care in each country and optimize future decisions by the headache community in each region. Additionally, this basic information about clinical practice will be beneficial in facilitating international discussions on headache care.

This novel study about CGRPmAb use in Japan has several strengths. The survey was sent only to members of the JHS, which ensured the quality of each response and limited the respondents to physicians. The survey was conducted entirely online, enabling us to reach physicians all over Japan. Some study limitations should be considered; only approximately 13% of the physicians in the JHS answered the survey. We suspect that our survey responses were exclusively from physicians who are treating patients with migraine. In addition, the respondents may have included several physicians from the same clinic/hospital, which may have affected the results about multiple CGRPmAb uses and the availability of CGRPmAbs at the different types of facilities. While our survey was conducted solely in Japanese, this manuscript was written in English; the results should be interpreted carefully due to potential but minimized linguistic discrepancy.

## Conclusions

We analyzed the responses to an online survey from 397 JHS members. The responses indicated that 320 of the physicians have experience of prescribing CGRPmAbs. Most of the respondents recommend CGRPmAbs to patients with ≥ 2 preventive failures, followed by ≥ 1. The MMD threshold ranged mostly from ≥ 4 to ≥ 10. The cost of CGRPmAbs is of great concern, since it was a frequently mentioned issue concerning CGRPmAbs and was also a frequently reported reason for responders to discontinue treatment with a CGRPmAb.

### Supplementary Information

**Additional file 1: Suppl. Table S1.** Questionnaire on headache practice and CGRPmAb usage. CGRPmAb: anti-calcitonin gene-related peptide monoclonal antibody, CM: chronic migraine, EM: episodic migraine, JHS: Japanese Headache Society, min: minute, MMD: monthly migraine days, yrs: years, No.: number.

**Additional file 2: Suppl. Table S2. **CGRPmAb availability at each type of facility. CGRPmAb: anti-calcitonin gene-related peptide monoclonal antibody, No.: number.

**Additional file 3: Suppl. Table S3. **Number of migraine preventives before a CGRPmAb is prescribed, and the MMD threshold for prescribing a CGRPmAb. MMD: monthly migraine days, CGRPmAbs: anti-calcitonin gene-related peptide monoclonal antibodies, No.: number.

## Data Availability

The datasets analysed during the current study are available from the corresponding author on reasonable requests.

## Abbreviations

CGRPmAbs: Anti-calcitonin gene-related peptide monoclonal antibodies
MMD: Monthly migraine days
JHS: Japanese Headache Society
EM: Episodic migraine
CM: Chronic migraine
SD: Standard deviation
ANOVA: Analysis of variance
RR: Responder rate
ICHD-3: International Classification of Headache Disorders, third edition
MIDAS: Migraine Disability Assessment
HIT-6: Headache Impact Test-6
MOH: Medication-overuse headache

## References

[1] Global, regional, and national burden of migraine and tension-type headache, 1990–2016: a systematic analysis for the Global Burden of Disease Study 2016 Lancet Neurol 2018;17(11):954-76. 10.1016/S1474-4422(18)30322-3 PMC6191530 30353868

[2] Sakai F, Igarashi H (1997). Prevalence of migraine in Japan: a nationwide survey. Cephalalgia.

[3] Goadsby PJ, Reuter U, Hallström Y, Broessner G, Bonner JH, Zhang F (2017). A Controlled Trial of Erenumab for Episodic Migraine. N Engl J Med.

[4] Skljarevski V, Matharu M, Millen BA, Ossipov MH, Kim BK, Yang JY (2018). Efficacy and safety of galcanezumab for the prevention of episodic migraine: Results of the EVOLVE-2 Phase 3 randomized controlled clinical trial. Cephalalgia.

[5] Dodick DW, Silberstein SD, Bigal ME, Yeung PP, Goadsby PJ, Blankenbiller T (2018). Effect of Fremanezumab Compared With Placebo for Prevention of Episodic Migraine: A Randomized Clinical Trial. JAMA.

[6] Sakai F, Ozeki A, Skljarevski V. Efficacy and safety of galcanezumab for prevention of migraine headache in Japanese patients with episodic migraine: A phase 2 randomized controlled clinical trial. Cephalalgia Reports. 2020;3.

[7] Takeshima T, Sakai F, Hirata K, Imai N, Matsumori Y, Yoshida R (2021). Erenumab treatment for migraine prevention in Japanese patients: Efficacy and safety results from a Phase 3, randomized, double-blind, placebo-controlled study. Headache.

[8] Sakai F, Suzuki N, Kim BK, Tatsuoka Y, Imai N, Ning X (2021). Efficacy and safety of fremanezumab for episodic migraine prevention: Multicenter, randomized, double-blind, placebo-controlled, parallel-group trial in Japanese and Korean patients. Headache.

[9] Sakai F, Suzuki N, Kim BK, Igarashi H, Hirata K, Takeshima T (2021). Efficacy and safety of fremanezumab for chronic migraine prevention: Multicenter, randomized, double-blind, placebo-controlled, parallel-group trial in Japanese and Korean patients. Headache.

[10] Takizawa T, Ohtani S, Watanabe N, Miyazaki N, Ishizuchi K, Sekiguchi K (2022). Real-world evidence of galcanezumab for migraine treatment in Japan: a retrospective analysis. BMC Neurol.

[11] Suzuki K, Suzuki S, Shiina T, Tatsumoto M, Fujita H, Haruyama Y (2023). Effectiveness of three calcitonin gene-related peptide monoclonal antibodies for migraine: A 12-month, single-center, observational real-world study in Japan. Cephalalgia.

[12] Sacco S, Amin FM, Ashina M, Bendtsen L, Deligianni CI, Gil-Gouveia R (2022). European Headache Federation guideline on the use of monoclonal antibodies targeting the calcitonin gene related peptide pathway for migraine prevention - 2022 update. J Headache Pain.

[13] Hong JB, Lange KS, Fitzek M, Overeem LH, Triller P, Siebert A (2023). Impact of a reimbursement policy change on treatment with erenumab in migraine - a real-world experience from Germany. J Headache Pain.

[14] American HS (2019). The American Headache Society Position Statement On Integrating New Migraine Treatments Into Clinical Practice. Headache.

[15] GoodRx. http://www.goodrx.com Accessed Oct. 13, 2023.

[16] Pascual J, Nunez M, Panni T, Diaz-Cerezo S, Novick D, Ciudad A (2023). Burden and Unmet Needs in Migraine Patients: Results from the OVERCOME (Spain) Study. Pain Ther.

[17] National Health Insurance Administration. Ministry of Health and Welfare, Taiwan. National Health Insurance in Taiwan. https://eng.nhi.gov.tw/en/mp-2.html. Accessed 13 Oct 2023.

[18] Ihara K, Ohtani S, Watanabe N, Takahashi N, Miyazaki N, Ishizuchi K (2023). Predicting response to CGRP-monoclonal antibodies in patients with migraine in Japan: a single-centre retrospective observational study. J Headache Pain.

[19] Barbanti P, Aurilia C, Egeo G, Torelli P, Proietti S, Cevoli S (2023). Late Response to Anti-CGRP Monoclonal Antibodies in Migraine: A Multicenter Prospective Observational Study. Neurology.

[20] National Tax Agency. Statistical Survey of Actual Status for Salary in the Private Sector. https://www.nta.go.jp/publication/statistics/kokuzeicho/minkan/gaiyou/2022.htm. (in Japanese) Accessed on Dec. 23, 2023.

[21] Sacco S, Lampl C, Maassen van den Brink A, Caponnetto V, Braschinsky M, Ducros A (2021). Burden and attitude to resistant and refractory migraine: a survey from the European Headache Federation with the endorsement of the European Migraine & Headache Alliance. J Headache Pain.

