# Supplementary material for: CGRP-monoclonal antibodies in Japan: insights from an online survey of physician members of the Japanese headache society
Source: J Headache Pain. 2024 Mar 15;25(1):39. doi: 10.1186/s10194-024-01737-y (PMC10941476; doi:10.1186/s10194-024-01737-y)
Supplement: Supplementary file 1 — Additional file 1: Suppl. Table S1. Questionnaire on headache practice and CGRPmAb usage. CGRPmAb: anti-calcitonin gene-related peptide monoclonal antibody, CM: chronic migraine, EM: episodic migraine, JHS: Japanese Headache Society, min: minute, MMD: monthly migraine days, yrs: years, No.: number. [file 10194_2024_1737_MOESM1_ESM.docx]

**Suppl. Table S1**

| **Question** | **Answer** | **Section** | |
| --- | --- | --- | --- |
| Any experience using CGRPmAbs | Yes → Section 2 No → Section 3 | 1 | |
| Facility | Clinic Community hospital University hospital | 2  2 | 3 |
| Service field | Neurology Neurosurgery Internal medicine (other than neurology) Pediatrics Anesthesiology/Pain clinic Others |  |  |
| Age, yrs | 20s 30s 40s 50s ≥60s |  |  |
| Years of headache practice | <5 yrs ≥5 and <10 yrs ≥10 and <20 yrs ≥20 yrs |  |  |
| Years of JHS membership | <1 yrs ≥1 and <5 yrs ≥5 and <10 yrs ≥10 yrs |  |  |
| sBoards | Headache Neurology Neurosurgery Internal Medicine |  |  |
| Length of the first appointment of headache patients, min |  |  |  |
| Length of the follow-up appointments of headache patients, min |  |  |  |
| No. of patients with migraine who are followed up regularly |  |  |  |
| No. of patients with migraine who are followed up regularly and have ever used migraine preventive treatments |  |  |  |
| No. of patients with migraine who are followed up regularly and have ever used CGRPmAbs |  |  |  |
| CGRPmAbs that are available in your facility | Galcanezumab Erenumab Fremanezumab None of the above |  |  |
| The MMD threshold for recommending CGRPmAbs | ≥4 ≥6 ≥8 ≥10 ≥12 ≥15 |  |  |
| No. of migraine preventives you usually try before prescribing a CGRPmAb | 1 2 3 4 ≥5 |  |  |
| When to assess the response to CGRPmAbs for patients with EM | After 1 month After 2 months After 3 months After 4–6 months After 7–9 months After 10–12 months |  |  |
| When to assess the response to CGRPmAbs for patients with CM | After 1 month After 2 months After 3 months After 4–6 months After 7–9 months After 10–12 months |  |  |
| The percentage of EM patients whose MMDs have decreased ≥50% | <20% ≥20% and <40% ≥40% and <60% ≥60% and <80% ≥80% |  |  |
| The percentage of CM patients whose MMDs have decreased ≥50% | <20% ≥20% and <40% ≥40% and <60% ≥60% and <80% ≥80% |  |  |
| The most frequently reported reason for responders to discontinue CGRPmAbs | Cost Adverse effects, safety (including injection site reaction, constipation, pregnancy) Frequent hospital visits Enough improvement of migraine Others |  |  |
| Any requests about CGRPmAbs |  |  | 3 |
