# Supplementary material for: CGRP-monoclonal antibodies in Japan: insights from an online survey of physician members of the Japanese headache society
Source: J Headache Pain. 2024 Mar 15;25(1):39. doi: 10.1186/s10194-024-01737-y (PMC10941476; doi:10.1186/s10194-024-01737-y)
Supplement: Supplementary file 2 — Additional file 2: Suppl. Table S2. CGRPmAb availability at each type of facility. CGRPmAb: anti-calcitonin gene-related peptide monoclonal antibody, No.: number. [file 10194_2024_1737_MOESM2_ESM.docx]

**Suppl. Table S2**

| **Facility** | **No. of CGRPmAbs** | | | | **Availability** | | | **Total** |
| --- | --- | --- | --- | --- | --- | --- | --- | --- |
|  | **0** | **1** | **2** | **3** | **Erenumab** | **Galcanezumab** | **Fremanezumab** |  |
| Community hospital | 24 (12.4%) | 52 (26.8%) | 39 (20.1%) | 79 (40.7%) | 101 (52.1%) | 154 (79.4%) | 112 (57.7%) | 194 (48.9%) |
| Clinic | 8 (6.4%) | 13 (10.3%) | 20 (15.9%) | 85 (67.5%) | 93 (73.8%) | 112 (88.9%) | 103 (81.8%) | 126 (31.7%) |
| University hospital | 2 (2.6%) | 15 (19.5%) | 17 (22.1%) | 43 (55.8%) | 50 (64.9%) | 71 (92.2%) | 57 (74.0%) | 77 (19.4%) |
| Total | 34 (8.6%) | 80 (20.2%) | 76 (19.1%) | 207 (52.1%) | 244 (61.5%) | 337 (84.9%) | 272 (68.5%) | 397 |
