# Supplementary material for: CGRP-monoclonal antibodies in Japan: insights from an online survey of physician members of the Japanese headache society
Source: J Headache Pain. 2024 Mar 15;25(1):39. doi: 10.1186/s10194-024-01737-y (PMC10941476; doi:10.1186/s10194-024-01737-y)
Supplement: Supplementary file 3 — Additional file 3: Suppl. Table S3. Number of migraine preventives before a CGRPmAb is prescribed, and the MMD threshold for prescribing a CGRPmAb. MMD: monthly migraine days, CGRPmAbs: anti-calcitonin gene-related peptide monoclonal antibodies, No.: number. [file 10194_2024_1737_MOESM3_ESM.docx]

**Suppl. Table S3**

| **MMD threshold for  prescribing CGRPmAbs** | **No. of migraine preventives before CGRPmAbs** | | | | | |
| --- | --- | --- | --- | --- | --- | --- |
|  | **1** | **2** | **3** | **4** | **≥5** | **Total** |
| ≥4 | 26 (8.3%) | 36 (11.5%) | 8 (2.6%) | 0 (0%) | 1 (0.3%) | 71 (22.8%) |
| ≥6 | 16 (5.1%) | 39 (12.5%) | 10 (3.2%) | 3 (1.0%) | 0 (0%) | 68 (21.8%) |
| ≥8 | 10 (3.2%) | 40 (12.8%) | 22 (7.1%) | 3 (1.0%) | 1 (0.3%) | 76 (24.4%) |
| ≥10 | 10 (3.2%) | 48 (15.4%) | 15 (4.8%) | 5 (1.6%) | 3 (1.0%) | 81 (26.0%) |
| ≥12 | 1 (0.3%) | 2 (0.6%) | 1 (0.3%) | 0 (0%) | 0 (0%) | 4 (1.3%) |
| ≥15 | 1 (0.3%) | 5 (1.6%) | 6 (1.9%) | 0 (0%) | 0 (0%) | 12 (3.8%) |
| Total | 64 (20.5%) | 170 (54.5%) | 62 (19.9%) | 11 (3.5%) | 5 (1.6%) | 312 (100%) |
